# Father involvement, maternal depression and child nutritional outcomes in Soweto, South Africa

**DOI:** 10.1111/mcn.13177

**Published:** 2021-07-09

**Authors:** Roisin E. Drysdale, Wiedaad Slemming, Tawanda Makusha, Linda M. Richter

**Affiliations:** ^1^ DSI‐NRF Centre of Excellence in Human Development University of the Witwatersrand Johannesburg South Africa; ^2^ Division of Community Paediatrics, Department of Paediatrics and Child Health, Faculty of Health Sciences University of the Witwatersrand Johannesburg South Africa; ^3^ Human Sciences Research Council Pretoria South Africa; ^4^ Wits/MRC Developmental Pathways for Research Unit Johannesburg South Africa

**Keywords:** breastfeeding, depression, fathers, low birth weight, maternal mental health, maternal public health, pregnancy, South Africa

## Abstract

Father involvement in South Africa is low, despite evidence that it can improve maternal and child health and wellbeing. Within a larger randomised controlled trial, we assessed whether father involvement during and after pregnancy increased birth weight and exclusive breastfeeding through improved maternal mental health. At 6‐week postnatal, mothers completed questionnaires on birth, feeding practices, social support, father involvement and postnatal depression. Father involvement during pregnancy was measured by their attendance at antenatal care and the study intervention, whereas postnatal involvement was measured by attendance at antenatal care and type of paternal support provided. Structural equation modelling was used to identify associations between father involvement, maternal depression, low birth weight and exclusive breastfeeding. Among the 212 mother–baby pairs, father involvement was very low with only 43%, 33% and 1% of partners attending early ultrasound, antenatal care and the birth of the child, respectively. Twenty‐nine percent of the mothers showed signs of depression during pregnancy, compared with 7% after birth. Eighteen percent of the infants were born low birth weight, and 57% of mothers reported exclusively breastfeeding at 6 weeks. Father involvement was directly associated with postnatal depression, but it did not directly or indirectly impact exclusive breastfeeding or low birth weight. We conclude that postnatal father involvement can improve postnatal maternal depression and that men would benefit from specific guidance on how they can support mothers during and after pregnancy.

**Key messages**

- Father attendance at pregnancy ultrasound was low despite a personal invitation and the service being offered on a weekend.
- Structural barriers within the public health system in South Africa continue to limit male attendance at the birth of their child.
- Father involvement during and after pregnancy was associated with improved maternal mental health.
- Interventions to improve male involvement should provide men with specific information and guidance on how best to support their partners during and after pregnancy.
- Research is needed to assess the long‐term impacts of father involvement on maternal wellbeing and healthy child growth and development.

## 1 INTRODUCTION

The first 1,000 days of life, from conception to a child's second birthday, are a time of both tremendous potential and vulnerability (Richter et al., 2017). During this time, a child's brain grows more quickly than any other time, and health, nutrition, protection, and responsive care during pregnancy and infancy fundamentally affect a child's growth and development (Black et al., 2017; Prado & Dewey, 2014). Maternal undernutrition is a risk for preterm birth and low birth weight (Accrombessi et al., 2017; Blencowe et al., 2019), which both increase the risks of stunting and long‐term developmental challenges (Blencowe et al., 2013; Christian et al., 2013; Gluckman et al., 2006). Worldwide, approximately 15% of infants are born of low birth weight [World Health Organization (WHO), 2014a], and although there has been some improvement in the prevalence of low birth weight, the progress made is slower than required to meet either the Global Nutrition Targets (Blencowe et al., 2019; WHO, 2014b, 2018) or the Sustainable Development Goals (UNICEF, 2020). At the same time, child malnutrition remains a global challenge with 159 million children under five stunted and 49.5 million wasted (Global Nutrition Report, 2020). Research has indicated that exclusive breastfeeding for the first 6 months can reduce childhood undernutrition and simultaneously improve protection from infectious diseases and the associated adverse nutritional effects (Scherbaum & Srour, 2016). Despite this, in 2017, only 41% of infants aged between 0 and 5 months were exclusively breastfed globally; an increase of only 4% on the previous 5 years (Global Nutrition Report, 2020).

Nurturing care is key for optimal child development and growth both during and after pregnancy (Britto et al., 2016; Cusick & Georgieff, 2016; Schwarzenberg & Georgieff, 2018) with families and others playing a key role in reducing stress, providing information and practical or financial support. Evidence suggests that mothers with low levels of social support during early pregnancy are more likely to have babies with low birth weight (Elsenbruch et al., 2007), and mothers benefiting from emotional support from their partners, family or friends are less likely to suffer postpartum complications (Maharlouei, 2016). Family members can positively influence and support feeding practices, but also undermine feeding decisions made by the mother (Mphego et al., 2014; Raj & Plichta, 1998; Trafford et al., 2020). In traditional societies, women often face pressure from grandmothers, mothers or sisters to give solid food, water, formula or traditional medicine before an infant turns 6 months old (Mphego et al., 2014).

The role of men in the perinatal period is often neglected, but their involvement during pregnancy and early childhood can lead to improved health and developmental outcomes at birth and throughout a child's life (Feldman et al., 2013; Jackson, 2017; Lamb, 2010; McKee et al., 2018; Walsh et al., 2017). During pregnancy, fathers often provide financial, practical and emotional support to their pregnant partner (Plantin et al., 2011), and they can also promote positive maternal behaviours such as healthier eating, reduced smoking or drinking, and earlier attendance at and adherence to antenatal and postnatal health care (Alio et al., 2010; Makusha & Richter, 2018; Martin et al., 2007). In contrast, less father involvement during pregnancy has been found to be associated with low birth weight, small for gestational age (Alio et al., 2010) and preterm birth (Ghosh et al., 2010), all of which are associated with lifelong elevated risk for cardiovascular disease (Souza et al., 2019), hypertension and diabetes (Kibret et al., 2019; Knop et al., 2018). Lack of father involvement in infant and young feeding has been found to be associated with undernutrition in children (Kansiime et al., 2017), and studies have reported that if a women's partner approves of their breastfeeding, they are three times more likely to exclusively breastfeed than those whose partners don not approve (Perez‐Escamilla, 1994). In sum, engaged and informed men can encourage optimum feeding practices such as exclusive breastfeeding and appropriate complimentary feeding (Dinga et al., 2018; Maycock et al., 2013), both of which lead to improved child health, nutrition and development outcomes (Kramer & Kakuma, 2012; WHO, 2020).

In addition to improved birth and child development outcomes, father involvement can have a profound impact on the mental health of women. Mothers with partners who are highly involved during pregnancy have been found to report lower levels of antenatal depression (Cheng et al., 2016; Maselko et al., 2019), whereas lower partner involvement postnatally increases the risk of postnatal depression (Lin et al., 2017). Research suggests that antenatal depression can negatively impact foetal development and increase the risk of preterm or still birth as well as low birth weight (Brittain et al., 2015; Dadi, Miller, & Mwanri, 2020; Dadi, Miller, Woodman, et al., 2020), and postnatal depression has been associated with reduced exclusive breastfeeding (Silva et al., 2017). In this regard, increased father involvement can positively affect child birth and developmental outcomes indirectly through improved maternal mental health (Dadi, Miller, Woodman, et al., 2020; Maselko et al., 2019).

In South Africa, despite the evidence and calls from health professionals (McKee et al., 2018), there are a number of health system, individual, social and structural barriers that prevent men from being actively involved in antenatal care, the birth of their child or early childcare. These include poorly structured or under‐resourced health services, limited clinic hours, negative attitudes among health care workers, low marriage and cohabitation rates, low father–child residency, and high rates of migrant labour, unemployment and poverty (Hastings‐Tolsma et al., 2018; Makusha & Richter, 2018). At the same time, women face a range of social and economic issues that contribute to poor mental health, including high rates of gender‐based violence, crime, poverty and food insecurity (Perry et al., 2010; Van Heyningen et al., 2017).

The negative impact of these barriers is manifested in recent figures that suggest that 15% of births in South Africa are low birth weight and only 32% of babies under 6 months of age are exclusively breastfed [National Department of Health (NDoH), 2019]. In terms of maternal depression, estimates in South Africa vary depending on the measure used, time of measurement and characteristics of the sample (Pellowski et al., 2019), but range between 21% and 50% (Brittain et al., 2015; Peltzer et al., 2018; Van Heyningen et al., 2016). Lack of improvement on these indicators highlights that additional efforts to improve both maternal and child health outcomes are required in South Africa.

In order to improve father involvement during the antenatal and postnatal period, we invited the partners of pregnant women to attend a pregnancy ultrasound, part of an antenatal intervention to improve child growth and development. In the postnatal follow up of the intervention at 6 weeks, we assessed whether father involvement during and after pregnancy is associated with improved birth weight and exclusive breastfeeding in an urban setting in South Africa. We hypothesised that these relationships are mediated by maternal mental health.

## 2 METHODS

### 2.1 Study design

This paper reports on the 6‐week follow up from a randomised controlled trial assessing the impact of augmented ultrasound during pregnancy on healthy child growth and development compared with standard practice of care. Recruited participants were blindly allocated to the intervention or control arms. A routine ultrasound scan was offered to all participants in the study. The ultrasound examination in the control group followed standard practice of care, whereas the intervention group received additional messages to promote early childhood development, an educational baby book and a printed and electronic image of their ultrasound scan. Full details of the trial intervention and methods can be found in Richter et al. (2020).

### 2.2 Setting

The study is situated in Soweto, South Africa's largest township located near Johannesburg. All study procedures took place between March 2019 and August 2020 in a research unit at Chris Hani Baragwanath Hospital (CHBH), a tertiary hospital in Soweto.

### 2.3 Participants

Participants were recruited from the Foetal Medicine Unit (FMU) at CHBH and invited to participate if they had a singleton pregnancy <25 weeks gestation and were over the age of 18 years and lived within Soweto. Women who were found to have severe maternal comorbidities or major foetal abnormalities were excluded from the study but remained in clinical care at CHBH. Participants who attended the ultrasound were requested to attend follow‐up visits with their infant 6 weeks after birth. Mothers in both the intervention and control groups were encouraged to invite the father of the baby to attend all study appointments and were provided with invitation cards to give to their male partner on recruitment.

### 2.4 Data collection

During the ultrasound visit, women completed individual questionnaires on their sociodemographic characteristics and relationship status with the father of the baby. Antenatal depression was measured using the Edinburgh Postnatal Depression Scale (EPDS), a 10‐item self‐reported depression scale that has been validated for use during the antenatal period (Choi et al., 2012) and in South Africa (Lawrie et al., 1998). As per recommendations by Cox et al. (1987), mothers with a score of ≥10 were referred to a specialised nurse for further assessment and to receive counselling if required.

At the 6‐week follow‐up visit, the mothers were asked questions related to the birth of their baby, including the type of delivery and complications experienced by the mother or baby, as well as questions on their feeding practices, such as when the baby was put to the breast after birth. Mothers were asked if they had given their baby formula, water or porridge, and also asked to list all the foods and liquids they had given their baby up until the time of the interview. Those who did not report giving formula, water, porridge or other substances were recorded as having exclusively breastfed up to 6 weeks. To limit the overestimation of infants exclusively breastfed at 6 weeks, we chose not to follow the WHO infant and young child feeding guidelines that use a 24‐h recall method (WHO, 2008). Mothers were also asked about partner support and involvement during and after their pregnancy, specifically men's attendance at health checks or the birth, and the types of support partners were providing. Finally, mothers completed the EPDS for an assessment of postnatal depression and were referred for further assessment with a score of ≥10. Details of the infant, including their date of birth and birth weight, were taken from the Road to Health Book that is maintained for all children from birth in South Africa to keep track of their health, growth and development.

An index of socio‐economic status (SES) was calculated using a principal component analysis (PCA) based on data collected at baseline. Variables included in the index were crowding (number of rooms used for sleeping), household water and sanitation, internet access, and ownership of a washing machine, satellite television, DVD player, computer and car. The index was divided into tertiles. Antenatal father involvement was based on whether the partner attended the study ultrasound or additional antenatal health visits and was recorded as none, at least one or both. Postnatal father involvement was measured using a cumulative score comprising different forms of father involvement, such as responsibility or provisioning as per recommendations from Greene et al. (2001). This included whether the father had provided information, practical, emotional or financial support, was rated by the mother as providing her with the most help with the baby or her own health and how she rated this help, whether he made things harder for the mother by his behaviour or attitude and his attendance at antenatal health visits. We opted to use maternal reports of father involvement as perceived support is important, and we anticipated that father attendance at 6‐week follow‐up would be low. Low birth weight was classified as <2.5 kg. Binary variables were coded using 1 and 0 for yes and no responses, respectively.

### 2.5 Data analysis

Data were captured and analysed using Stata I/C 14. Categorical descriptive statistics are presented as frequencies and proportions. Continuous descriptive statistics are presented as minimum, maximum, mean and standard deviations.

**FIGURE 1:**
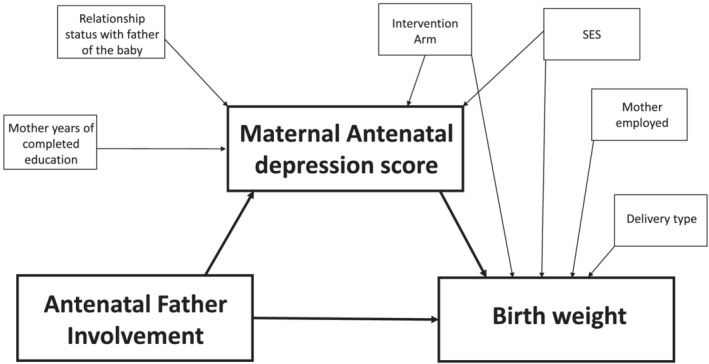
Pathway diagram for Model 1: antenatal father involvement, antenatal maternal mental health and birth weight. SES, socio‐economic status

**FIGURE 2:**
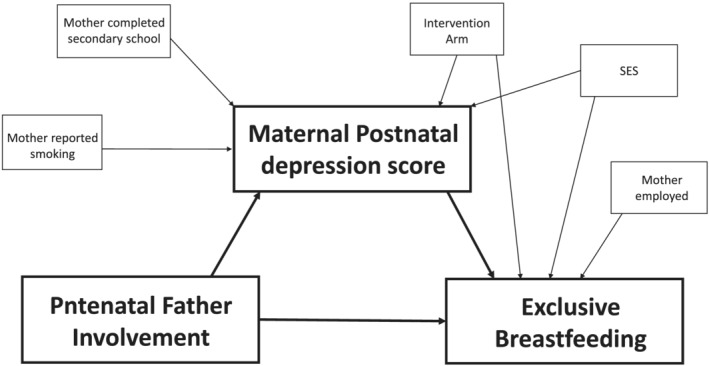
Pathway diagram for Model 2: father involvement, postnatal maternal mental health and exclusive breastfeeding. SES, socio‐economic status

Using structural equation modelling (SEM), two individual models were built to test our hypotheses; Model 1 assessed whether antenatal father involvement improved birth weight by improving antenatal maternal mental health, and Model 2 assessed whether father involvement during and after pregnancy increased exclusive breastfeeding through improved postnatal mental health. Antenatal variables were used in Model 1, whereas a combination of antenatal and postnatal variables was used in Model 2. Simple linear regression was undertaken to identify potential confounding variables for antenatal and postnatal maternal mental health. Variables with a *p* value < 0.10 were included in the SEM. For the main outcome variables (birth weight and exclusive breastfeeding), simple linear and univariate logistic regression, respectively, was conducted with all potential confounding maternal variables included in the SEM if the *p* value was <0.10. Intervention arm was controlled for both models. Figures 1 and 2 show the path diagrams used to guide each of the models. Due to the differing types of outcome data (continuous and binary), the results of the SEM are presented as total, direct and indirect *β* coefficients. We assess the fit of each model using the root square mean error of approximation (RMSEA), the comparative fit index (CFI) and the Tucker–Lewis index (TLI). A well‐fitting model has RMSEA < 0.05, and CFI and TLI > 0.95. All statistical tests were considered significant with a *p* value of <0.05, and 95% confidence intervals are presented.

### 2.6 Sample size

The required sample size for the trial was 100 participants per arm. In total, 249 pregnant women were enrolled in the trial, with 212 attending the 6‐week follow up. We conducted a reverse power analysis using G*power to determine whether the sample size of 212 was sufficient to conduct an SEM analysis. Based on an effect size of 0.17, alpha of 0.05 and two predictors, the power was 0.99 and the sample size sufficient for analysis.

### 2.7 Ethical considerations

The study was approved by the Human Research Ethics Committee (Medical) of the University of the Witwatersrand, South Africa (M181915). An amendment was approved by the University of the Witwatersrand in order to film some of the ultrasound appointments. Permission was obtained from CHBH to conduct the study and to oversee adherence to the study protocol. The trial is registered with the Pan African Clinical Trials Registry (PACTR201808107241133). Participants provided written consent and were given a unique study identifier to maintain confidentiality.

## 3 RESULTS

Out of the 249 mothers who attended the ultrasound appointment at baseline, 12 of their babies died within their first 6 weeks of life, and 25 were lost‐to‐follow‐up. Presented results are based on the 212 mother–baby pairs who attended the 6‐week follow‐up visit.

### 3.1 Participant characteristics

The sociodemographic characteristics of the participants at baseline are shown in Table 1. The mean age of mothers' was 32 years (SD = 0.45), ranging from 18 to 47 years. The mean age of fathers' was 36 years (SD = 0.50), ranging from 20 to 56 years. A third of mothers (64%) had completed high school with the mean years at school being 11, the maximum 17 and the minimum 0. Forty‐five percent of the mothers were employed during pregnancy. Approximately, 90% of participants were either married or in a committed relationship with the father of their baby, although only 28% reported living with their partner. For 18% of mothers, this was their first pregnancy. Almost a third (30%) had had one previous pregnancy, with 29% and 12% having had two or three, respectively. For the mothers who had been pregnant before, 53% reported that they had breastfed their baby, either exclusively or mixed feeding. A small number of the mothers reported smoking (3%) and drinking alcohol (5%) throughout their pregnancy.

**TABLE 1:** Descriptive statistics

	*n*	%
**Maternal sociodemographic characteristics**		
Socio‐economic status		
Highest	73	31.43
Middle	70	33.02
Lowest	69	32.55
Mother completed secondary school		
Yes	135	63.7
No	77	36.3
Mother employed during pregnancy		
Yes	96	45.3
No	116	54.7
Relationship status with father of baby		
Not in a committed relationship	21	9.9
Married or committed relationship (living together)	60	28.3
Married or committed relationship (not living together)	130	61.3
Refuse to answer	1	0.5
Mother has other children		
Yes	174	82.1
No	38	17.9
Mother breastfeed previous child		
Yes	92	52.9
No	82	47.1
Mother reported smoking during pregnancy		
Yes	7	3.30
No	205	96.70
Mother reported drinking alcohol during pregnancy		
Yes	11	5.19
No	201	94.81
**Birth information and feeding practices**		
Delivery type		
Normal vaginal delivery	100	47.17
Caesarean section	112	52.83
Mother experienced complications at birth		
Yes	34	16.04
No	178	83.96
Baby experienced complications at birth		
Yes	30	14.15
No	182	85.85
Mother put baby to breast within 1 h after birth		
Yes	120	56.60
No	92	43.40
Exclusive breastfeeding at 6 weeks		
Yes	121	57.08
No	91	42.94
Low birth weight (<2.5 kg)		
Yes	38	18.36
No	169	81.64

### 3.2 Child feeding and birth information

Mode of delivery was almost evenly distributed between normal vaginal delivery and caesarean section at 47% and 53%, respectively. Sixteen percent of mothers and 14% of babies reported experiencing complications at birth, respectively. Complications for the mother included excessive bleeding (9%), hypertension (27%) and infection (35%). Complications for the baby included preterm birth (27%), respiratory difficulties (17%) and jaundice (20%). Eighteen percent of babies were born low birth weight and the mean, minimum and maximum birth weight was 2.96 (SD = 0.57), 0.93 and 4.38 kg, respectively. Just over half (57%) of mothers reported that they put their baby to the breast within an hour after birth and by 6 weeks, 57% of the mothers were exclusively breastfeeding.

### 3.3 Father involvement

As seen in Table 2, fewer than half (43%) of male partners attended the study in United States with lower attendance (33%) of at least one routine antenatal care visit. Only 19% of the partners attended both the study ultrasound and at least one routine antenatal visit, whereas 37% attended one or the other, and 44% attended neither. Only 1% of partners were present at the birth of their child. Reasons given for fathers not attending their child's birth included hospital staff restricting their access (60%), the father working or living at a distance (28%), and the father being at work (8%). When asked if they felt that their partner made things harder for them because of the way they acted, 71% stated never, but 18% and 11% stated sometimes or always, respectively. Just over half of the mothers (52%) reported that their partners gave the most help with their own health, whereas 33% stated that they gave the most help with the baby. The majority of the participants rated their partners help as good or very good (83%), but 7% reported it as fair and 10% as poor. The most common type of support provided by the fathers was financial, although this was still low at 30% and the least common type of support provided was information (19%). The maximum father involvement score was 14, with 4% of male participants achieving the highest score. The mean father involvement score was 8 (SD = 3.49), and 7% of men had the lowest score of one. The majority had a father involvement score between five and nine (54%), whereas 19% and 27% had scores below five and above 10, respectively.

**TABLE 2:** Father involvement

	*n*	%
Partner attended antenatal care		
Yes	69	32.55
No	143	67.45
Partner attended study ultrasound		
Yes	90	42.45
No	122	57.55
Partner attended birth		
Yes	3	1.42
No	209	98.58
Reason partner not present during childbirth		
He was not interested	3	1.44
He was at work	17	8.13
He works or lives away	57	27.27
The hospital would not let him	125	59.81
Other	7	3.35
Partner made things harder		
Always	24	11.32
Sometimes	37	17.45
Never	151	71.23
Partner gave the most help with the baby		
Yes	70	33.02
No	142	66.98
Partner gave most help with the mother		
Yes	111	52.36
No	101	47.64
Partner provided information		
Yes	41	19.34
No	171	80.66
Partner provided financial help		
Yes	63	29.72
No	149	70.28
Partner provided practical help		
Yes	61	28.77
No	151	71.23
Partner provided emotional support		
Yes	56	26.42
No	156	73.58
Mother rating of partner help		
Poor	22	10.38
Fair	14	6.60
Good	24	11.32
Very good	152	71.70

### 3.4 Maternal depression

Twenty‐nine percent of mothers scored more than 10 on the EPDS during pregnancy compared with 7% after birth. The mean antenatal depression score was seven (SD = 4.96), wereas the mean postnatal depression score was five (SD = 5.18). The maximum score on both occasions was 27.

### 3.5 Structural equation modelling

Table 3 shows the summarised results of the SEM for both models. Model 1 indicated that antenatal father involvement did not directly [*β*_direct_ = −0.0088, *p* = 0.865, 95% confidence interval (CI) = −0.11 to 0.09] or indirectly impact birth weight (*β*_indirect_ = −0.0018, *p* = 0.712, 95%CI = −0.01 to 0.01), nor did it directly affect antenatal maternal depression (*β*_direct_ = −0.1762, *p* = 0.700, 95%CI = −1.07 to 0.72). The model also indicated that antenatal maternal depression did not directly impact birth weight (*β*_direct_ = −0.0102, *p* = 0.186, 95%CI = −0.03 to 0.01). The results of the RMSEA and CFI suggested a well‐fitted model (RMSEA = 0.000, CFI = 1.00), but the TLI suggested a poorly fitted model (TLI = 0.411).

**TABLE 3:** Summary results of the structural equation modelling (SEM)

	Effect standardized coefficient (SE)			Model fit statistics		
	Total	Indirect	Direct	RMSEA	CFI	TLI
Model 1				0.000	1.000	1.411
Antenatal father involvement → Maternal antenatal depression score	−0.1762		−0.1762			
Maternal antenatal depression score → Birth weight	−0.0102		−0.0102			
Antenatal father involvement → Birth weight	−0.0070	−0.0018	−0.0088			
Model 2				0.000	1.000	1.429
Postnatal father involvement → Maternal postnatal depression score	−0.1578^*^		−0.1578^*^			
Maternal postnatal depression score → Exclusive breastfeeding	0.0144		0.0144			
Postnatal father involvement → Exclusive breastfeeding	0.0007	−0.0023	0.0030			

*Note*: Each model controlled for intervention arm. Model 1—associations between father involvement and depression controlled for SES, number of years mother completed education, and relationship with the father of the baby. Associations between father involvement and birth weight controlled for mother employment, SES and type of delivery. Model 2—associations between father involvement and depression controlled for SES, mother having completed secondary school and maternal smoking and alcohol behaviours. Associations between father involvement and exclusive breastfeeding controlled for mother employment and SES.

Abbreviations: CFI, comparative fit index; RMSEA, root square mean error of approximation; SES, socio‐economic status; TLI, Tucker–Lewis index.

* *p* < 0.05.

** *p* < 0.01.

The fit indexes for Model 2 fit the criteria for a well‐fitting model (RMSEA = 0.000, CFI = 1.000, TLI = 1.429). The results indicated that postnatal father involvement can directly impact maternal postnatal depression (*β*_direct_ = −0.1578, *p* = 0.019, 95%CI = −0.29 to −0.03) but that it does not indirectly (*β*_indirect_ = −0.0023, *p* = 0.208, 95%CI = −0.01 to 0.001) or directly (*β*_direct_ = 0.0030, *p* = 0.756, 95%CI = −0.02 to 0.02) influence exclusive breastfeeding. The results also indicated that postnatal depression did not directly influence whether a mother exclusively breastfed her infant (*β*_direct_ = 0.0144, *p* = 0.136, 95%CI = −0.01 to 0.03).

## 4 DISCUSSION

We hypothesised that father involvement could positively impact birth weight and exclusive breastfeeding at 6 weeks and that the relationship may be mediated by maternal mental health.

Although our study did not find that father involvement positively impacted birth weight or exclusive breastfeeding, we found that higher levels of father involvement after pregnancy were associated with better postnatal maternal mental health, similar to that found by Lin et al. (2017). Children require a nurturing and responsive environment to ensure optimal growth and development but poor mental health and depression in mothers can reduce their ability to recognise and respond appropriately to a child's needs, and affect how they express warmth, affection and pleasure (WHO et al., 2018). In addition, depression in mothers has been linked to stunting (Rahman et al., 2004) and long‐term adverse impacts on child development, including behavioural and mental health problems (Mezulis et al., 2004; Netsi et al., 2018). Therefore, it is vital to continuously involve fathers and encourage them to support their partners from conception and throughout childhood to reduce the negative impact poor mental health can have on both the mother and child.

That said, the results of the study highlight that male involvement during and after pregnancy and birth in South Africa is low and that a number of barriers to paternal support remain. Although the majority of the mothers did not rate their partners help poorly, they did indicate that it was not their partner who helped the most with the baby, and very few provided emotional, financial or practical support. We did not explore the reasons as to support was low, but research indicates this could include men working at a distance, unemployment, non‐fulfilment of cultural practices such as *inhlawulo* (acknowledgement of paternity) or *lobola* (bride price), poverty, housing shortages or incarceration (Makusha & Richter, 2018; Posel & Rudwick, 2014). The public health system can also act as a barrier with antenatal and postnatal services usually offered only during weekdays and working hours, which is a constraint for working men, particularly those in the informal industry. In addition, few public health facilities in South Africa accommodate a birthing companion, either male or female, due to staff attitudes or a lack of space, privacy concerns or resources (Brown et al., 2007; Lambert et al., 2018; Spencer et al., 2018). In this study, 60% of the women indicated that hospital staff denied their partner access to the birth, similar to experiences reported by Hastings‐Tolsma et al. (2018) and Malatji and Madiba (2020), also in South Africa. Although we offered the ultrasound appointment on a weekend to accommodate working men, very few utilised this option suggesting there could be additional social or cultural reasons for non‐attendance or low involvement, all of which should be explored further if male involvement is to be improved.

For the men that did attend the study ultrasound, after witnessing the scan, they reported positive feelings towards their partner and forthcoming child, as well as a desire to provide them with more support (Drysdale et al., 2020). As these results indicate, interventions that have targeted men directly and aimed to improve their involvement during the antenatal period have been shown to improve antenatal care attendance, maternal nutrition, birth preparedness and postpartum care. They can also improve communication and encourage joint decision‐making among couples (Tokhi et al., 2018). However, we also know that these interventions targeted at individual partners need to be supported by structural programmes, which include the uptake of paternity leave provisions for working fathers and creating a conducive health care system that accommodates both women and men during the perinatal period. By not targeting men, we may be missing an opportunity to improve child health, nutrition and development, as well as maternal health outcomes. Health services should incorporate messages and guidance specifically directed at men on how they can support women and children during and after pregnancy. These could include information on the importance of attending antenatal care, supporting their partners' adherence to antenatal supplements and the discouragement of substance use, and purchasing infant formula which men sometimes do to demonstrate their capacity to support their child, and preferably to support exclusive breastfeeding.

At 18%, the prevalence of low birth weight in this study was slightly higher than the 15% national average (NDoH, 2019). This may be attributable to the recruitment procedures of our study. Enrolled mothers were screened for foetal abnormalities and serious maternal health conditions. However, they were attending CHBH for antenatal care because they were deemed to be at risk during pregnancy due to comorbidities such as hypertension, diabetes or HIV. Research highlights that individual factors, such as maternal nutritional status, dietary practices and maternal behaviours such as smoking contribute to poor foetal growth and low birth weight (Bailey & Byrom, 2007; He et al., 2018; Kataoka et al., 2018; Rao & Raje, 2017; Sharma & Mishra, 2014). Based on this and our findings, we suggest that father involvement alone, especially in the context of low marriage and cohabitation rates and high unemployment and poverty, is not sufficient to improve birth weight. Nonetheless, as indicated by previous research, men can encourage and support women to practice healthy behaviours during pregnancy (Alio et al., 2010; Makusha & Richter, 2018; Martin et al., 2007), and specific guidance or advice on how to do so, as previously suggested, could help to reduce adverse birth outcomes.

In terms of feeding practices, 57% of the mothers were exclusively breastfeeding at 6 weeks, which is higher than the national average (NDoH et al., 2019). However, we anticipate that the rate of exclusive breastfeeding would fall by the time the infants are 6 months old. Early mother–infant separation remains one of the biggest barriers to exclusive breastfeeding in South Africa, mainly due to mothers returning to economic or educational activities (Horwood et al., 2020; Remmert et al., 2020) and a lack of supportive economic policies. For example, in South Africa, women working in the formal sector are entitled to a minimum of 4 months paid maternity leave claimed through the Department of Labour, which includes time prebirth and postbirth (Basic Conditions of Employment Act 75 of 1997). This is despite the NDoH recommendation to exclusively breastfeed up to 6 months and to continue breastfeeding up to 2 years (NDoH, 2013). Women working in the informal sector have no option but to return to work earlier. Although not apparent in our study, men can support and encourage breastfeeding if educated and informed (Dinga et al., 2018; Maycock et al., 2013) but without removing the aforementioned economic barriers in South Africa, exclusive breastfeeding will continue to be low in infants up to 6 months. The health and developmental benefits of early initiation and continued breastfeeding are conclusive (Hansen, 2016; Horta & Victoria, 2013a, 2013b; Kramer & Kakuma, 2012; Victoria et al., 2016; WHO, 2020); therefore, policies need to be reassessed and updated to ensure women receive the time, space and support to optimally feed their infants.

### 4.1 Study limitations

The study uses panel data collected at two time points to conduct a path analysis using SEM. Although this cannot determine causality, it is a useful approach to test our hypothesis and correlation between the variables of interest. As with many longitudinal studies, attrition occurred, and there was a loss to follow‐up rate of 14%: 4% due to death of the infant, and 10% due to mother withdrawal, relocation or inability to contact her. Due to limited information available, we are unable to determine whether father involvement or maternal mental health was in any way associated with infant death or loss to follow up. One strength of the study was the ability to take into account changes in mental health over time with repeated use of the EPDS; however, we did not take into account possible changes in sociodemographic characteristics, such as income or relationship status. In addition, antenatal father involvement was measured unidimensionally, which may explain the lack of associations between involvement and antenatal depression, particularly because more women showed signs of antenatal depression compared with postnatal depression. The data were collected from the female participants and is therefore missing valuable information on the fathers, including their employment or education status. In the 6‐month follow up, this information was collected from the men who attended the visit, but in future, it should be collected from baseline. Finally, the study assessed the impact of father involvement at birth and 6 weeks when the baby is very young, which may be too early to gauge any impact of father involvement. However, follow‐up is continuing to 6 months, and we plan to further assess whether father involvement influences child nutritional status and cognitive development at 6 months.

## 5 CONCLUSIONS

Our study found no association between father involvement, low birth weight or exclusive breastfeeding at 6 weeks; however, increased father involvement was associated with improved maternal postnatal mental health. Progress towards improving low birth weight and exclusive breastfeeding in South Africa remains slow and additional information, support and policy changes are required to effect large‐scale change. Men should be encouraged to attend antenatal health visits with their partner and should be provided with specific messages and guidance on how best to provide support during and after pregnancy. Given the South African context where more than two thirds of children reside in a household without their biological father, both public health and individual approaches will be needed. The overall message should be to encourage every father to be present, supportive and involved during and after pregnancy regardless of their relationship or residency status. Future research should conduct in‐depth analyses on barriers to father involvement and how these may impact maternal mental health.

## CONFLICTS OF INTEREST

The authors declare that they have no conflicts of interest.

## CONTRIBUTIONS

RED conceptualised the paper, conducted the analysis and wrote the first draft. WS, TM and LMR read and commented on the paper. LMR is the principal investigator of the Healthy Pregnancy Healthy Baby study. All authors read and approved the final version of the manuscript before submission.

## TRIAL REGISTRATION

The trial was registered through the Pan African Clinical Trials Registry in August 2018 (PACTR201808107241133).

## Data Availability

The data that support the findings of this study are available from the corresponding author upon reasonable request.
